# An Improved Machine Learning Approach for Optimizing Dust Concentration Estimation in Open-Pit Mines

**DOI:** 10.3390/ijerph20021353

**Published:** 2023-01-11

**Authors:** Boyu Luan, Wei Zhou, Izhar Mithal Jiskani, Zhiming Wang

**Affiliations:** 1 State Key Laboratory of Coal Resources and Safe Mining, China University of Mining and Technology, Xuzhou 221116, China; 2 School of Mines, China University of Mining and Technology, Xuzhou 221116, China; 3 Department of Mining Engineering, National University of Sciences & Technology, Balochistan Campus, Quetta 87300, Pakistan

**Keywords:** dust pollution, open-pit coal mine, estimation, random forest, Markov chains

## Abstract

Dust is a severe environmental issue in open-pit mines, and accurate estimation of its concentration allows for viable solutions for its control and management. This research proposes a machine learning-based solution for accurately estimating dust concentrations. The proposed approach, tested using real data from the Haerwusu open-pit coal mine in China, is based upon the integrated random forest-Markov chain (RF-MC) model. The random forest method is used for estimation, while the Markov chain is used for estimation correction. The wind speed, temperature, humidity, and atmospheric pressure are used as inputs, while PM2.5, PM10, and TSP are taken as estimated outputs. A detailed procedure for implementing the RF-MC is presented, and the estimated performance is analyzed. The results show that after correction, the root mean squared error significantly decreased from 7.40 to 2.56 μg/m^3^ for PM2.5, from 15.73 to 5.28 μg/m^3^ for PM10, and from 18.99 to 6.27 μg/m^3^ for TSP, and the Pearson correlation coefficient and the mean absolute error also improved considerably. This work provides an improved machine learning approach for dust concentration estimation in open-pit coal mines, with a greater emphasis on simplicity and rapid model updates, which is more applicable to ensure the prudent use of water resources and overall environmental conservation, both of which are advantageous to green mining.

## 1. Introduction

To cope with the growing severity of environmental challenges and climate change, in 2020, the Chinese government announced a new target toward a carbon dioxide emission peak by 2030. The country aims to strengthen regulations and take measures in order to attain carbon neutrality by 2060 [1]. One critical component of achieving a low-carbon future is expanding the usage of clean energy sources such as solar and wind [2]. However, in the case of an unstable clean energy supply and immature large-scale energy storage technologies, coal, China’s most abundant fossil fuel, is an essential source of energy security.

Due to the low safety and lower production efficiency challenges associated with underground mining, China’s open-pit coal mining capacity has increased year by year, from less than 5% of total coal output in 2000 to 10% in 2010 and 21% in 2016. At present, the proportion of open-pit mining also continues to grow [3]. However, open-pit mining brings a number of environmental problems and health issues [4,5], owing to the fact that green and climate-smart mining practices are not yet well implemented in China [6].

In relative terms, surface mining leads to more environmental degradation and air pollution than underground mining, with particulate matter pollution being one source of pollution [7,8], especially in northwest China, where rainfall is low, and water resources are scarce [9]. The high soil content and low rock hardness of the material stripped from open-pit coal mines make road dust a serious problem [10], especially in winter when air pressure is low [11]. As a result, significant watering is required to reduce the concentration of dust [12]. Therefore, as an indispensable component of green and climate-smart mining [13], the effective utilization of water resources has become an essential aspect of open-pit dust concentration control. The accurate prediction and estimation of dust concentrations are critical in this respect for optimizing water consumption rates.

Establishing monitoring stations is a highly practical method of obtaining environmental data, such as dust concentrations in open-pit mines [14]. Estimating dust concentrations based on measured data can effectively provide critical information that aids dust management in the area surrounding the monitoring locations. Researchers around the world have attempted to study dust concentration estimations. For instance, Tartakovsky, et al. [15] used the atmospheric dispersion models AERMOD and CALPUFF to estimate TSP in a quarry. The results showed that the estimations of AERMOD exhibited better agreement with the measurements than those of CALPUFF, while the estimations showed that on-site meteorological data proved to be the key to reliable dispersion calculations in complex terrain. Wanjun and Qingxiang [16] used a fluent model to simulate the transport pattern of dust concentration in an open-cast coal mine and analyzed the variation of dust concentration with the wind flow. Qi, et al. [17] used the particle swarm optimization method to optimize the random forest (RF) hyperparameters to improve dust concentration estimation accuracy. Lu, et al. [18] used a gradient boosting machine to predict dust concentration in an open-pit mine. Li, et al. [19] used long short-term memory (LSTM) to predict dust concentration in open-pit mines.

The literature demonstrates that, among the different tools and techniques available, machine learning—a multidisciplinary field method involving probability theory, statistics, and a range of other disciplines—is widely applicable and highly effective at predicting and estimating [20,21,22]. Previously, the research team had used the random forest-particle swarm optimization (RF-PSO) model to estimate dust concentrations in open-pit coal mines and achieved good results, with Pearson correlation coefficients of 0.91, RMSE of 6.97, and MAE of 3.95 for PM2.5 [17]. However, a serious problem was identified in subsequent studies. The problem is that the intensity of mining often changes during ongoing production due to a variety of factors, such as geological conditions, production schedules, and climate change, making it necessary to update the model frequently and over a relatively long period, while the expertise to update the model is lacking in Chinese open-pit coal mines. The direct cause of dust pollution and mining intensity can be added to the model inputs to reduce the frequency of updates. However, mining intensity is challenging to quantify, so a model with a low update time and low update difficulty is more suitable for field applications, as well as for existing mine personnel to learn and maintain the model.

This study, therefore, proposes the application of the random forest-Markov chain (RF-MC) model to dust concentration estimation, as it treats the transition of errors in the time-ordered dust concentration estimates as a Markov process and uses Markov chains to correct the estimations obtained through the RF model to improve the estimation accuracy. The core of this model update operation is the update of the MC model through historical data statistics, which allows for simple and fast model updates with the possibility of automatic updates through programming, making it more suitable for field applications. At the same time, this study is more concerned with the effect of Markov chains on the correction of random forests, especially for RF models with relatively poor estimation accuracy, which will affect the frequency of the RF model updates in the RF-MC model.

## 2. Materials and Methods

### 2.1. Study Area and Data Sources

**Figure 1:**
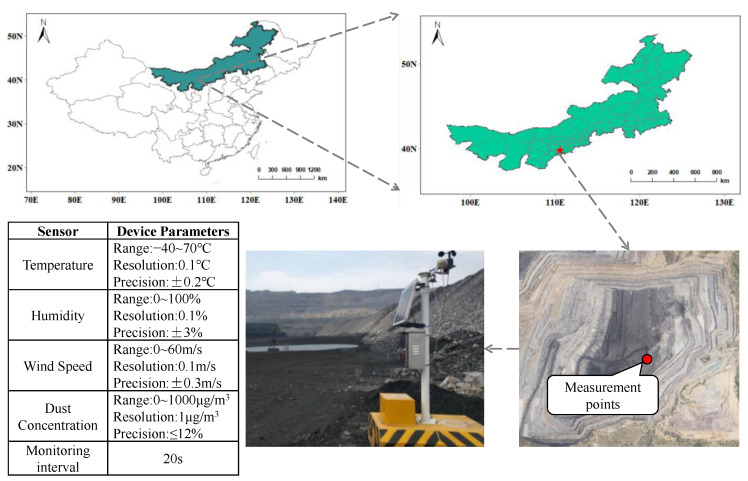
Mining area and measuring point location.

The Haerwusu open-pit coal mine in northwest China was chosen as the research location. The mine is located in a semi-arid region, belonging to a continental semi-dry climate, with low precipitation and high evaporation, requiring frequent watering of the haul roads. Figure 1 depicts the mining area, the location of the measuring points, and the parameters of the measuring device. The measuring equipment monitored PM2.5, PM10, TSP, temperature, humidity, wind speed, and atmospheric pressure every 20 s.

**Figure 2:**
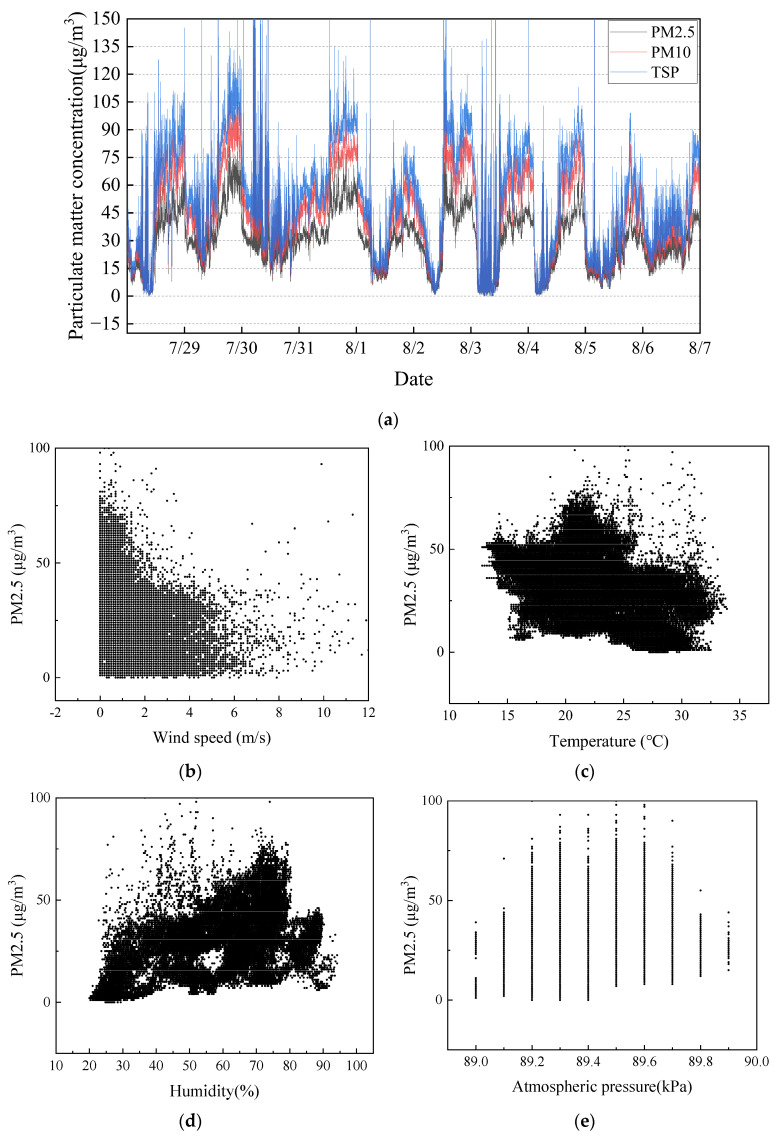
Measured data from 28 July 2020 to 7 August 2020: (**a**) dust concentration; (**b**) wind speed; (**c**) temperature; (**d**) humidity; (**e**) atmospheric pressure.

To ensure the statistical significance of the statistical results, a total of 41,381 sets of measured data were selected for this investigation over a ten-day period from 28 July to 7 August 2020. The weather was dry and hot during this period, with no rain. From the curves of PM2.5, PM10, and TSP over time, shown in Figure 2a, we can see that the changes in the three curves are basically the same and have a strong correlation. To observe the correlation between the data, PM2.5 and other characteristic variables were plotted in a scatter plot, as shown in Figure 2b. The scattering points between different characteristic variables and the dust concentration are more aggregated. The data has relatively few discrete data and shows a high correlation. The trend indicates that the data quality is sufficient to be used in machine learning.

### 2.2. Random Forest

**Figure 3:**
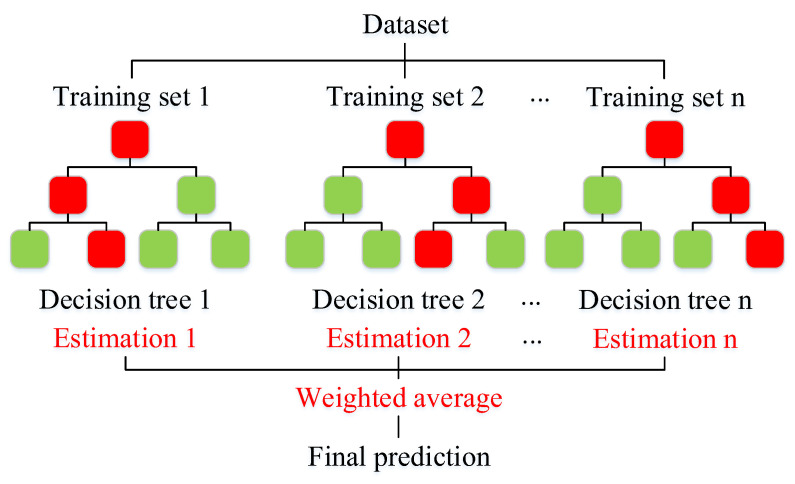
The architecture of random forest.

The RF algorithm, an effective machine learning prediction tool, was chosen to develop a model for dust concentration estimation. It is a non-parametric classification or regression technique belonging to a family of supervised machine learning techniques incorporating multiple decision trees to obtain the desired response [23]. The decision tree is a tree-structured decision algorithm where each leaf node represents a decision condition, and each branch represents a decision outcome. RF outperforms classical intelligence algorithms in terms of prediction accuracy and tolerance to outliers and noise [24]. Therefore, the reason for its selection is that the RF has a high noise tolerance. It can effectively find the optimal division of features on N random subsets of decision trees through weighted voting to address the shortcomings of decision trees, which are sensitive to individual data and overfit some datasets. Figure 3 illustrates the general architecture of an RF model.

### 2.3. Markov Chain

The Markov chain is a stochastic mathematical model in a finite time sequence describing a series of possible events in which the future state is solely related to the state of the preceding event [25]. The Markov property is a concept in probability theory, meaning that the evolution of the Markov process in the future depends only on the present state and does not depend on history [26,27]. In other words, a stochastic process has Markov properties if, given the present status, it is conditionally independent of the past status (i.e., the historical path of the process). A process with a Markov property is often called a Markov process. 

Since the previous dust concentration situation can determine the future dust concentration, dust concentration in the future can be seen as a Markov process. A Markov process in which both time and status are discrete is called a Markov chain, denoted as {*X_n_* = *X*(*n*), *n* = 0,1,2,...}, which can be seen as the result of successive observations of Markov processes with discrete statuses on time set *T*_1_ = {0,1,2,...}. The status space of the Markov chain is denoted as *I* = {*a*_1_, *a*_2_, ...}, *a_i_* ∈ R. In the case of chains, the Markov property is usually expressed in terms of the conditional distribution law, i.e., for any positive integer *n*, *r*, and 0 ≤ *t*_1_ < *t*_2_ < ... < *t_r_* < *m*; *t_i_*, *m*, *n* + *m* ∈ *T*_1_, the following equation can be derived.

(1) $$P\left\{X_{m+n}=\left.a_{j}\right|X_{t_{1}}=a_{i_{1}},X_{t_{2}}=a_{i_{2}},...,X_{t_{r}}=a_{i_{r}},X_{m}=a_{i}\right\}=P\left\{X_{m+n}=\left.a_{j}\right|X_{m}=a_{i}\right\}$$

where *a_i_* ∈ I. The right-hand side of the above equation can be written as the following equation:

(2) $$P_{ij}(m,m+n)=P\left\{\left.X_{m+n}=a_{j}\right|X_{m}=a_{i}\right\}$$

This is the transition probability of a Markov chain moving from status *a_i_* at moment *m*, to status *a_j_* at moment *m + n*.

Since a Markov chain departs from any status *a_i_* at moment *m* and necessarily transits to one of the many statuses *a_1_*, *a_2_*, etc., at another moment *m + n*, the following equation is derived:

(3) $$\sum_{j=1}^{+\infty }P_{ij}(m,m+n)=1,i=1,2,...$$

when the transition probability *P_ij_* (*m*, *m + n*) is only related to *i*, *j,* and the time step *n* (a Markov chain with stationary distribution), denoted as *P_ij_*(*n*), the following equation results.

(4) $$P_{ij}(m,m+n)=P_{ij}(n)$$

Moreover, the following equation is the n-step transition probability of a Markov chain.

(5) $$P_{ij}(n)=P\{X_{m+n}=a_{j}\left|X_{m}=a_{i}\}\right.$$

The n-step transition probability matrix can be obtained as:

(6) $$P(n)=\left[\begin{array}{cccc} p_{11} & p_{12} & ... & p_{1j}\\ p_{21} & p_{22} & ... & p_{2j}\\ \vdots & \vdots & \vdots & \vdots \\ p_{i1} & p_{i2} & ... & p_{ij} \end{array}\right]$$

### 2.4. Random Forest-Markov Chain (RF-MC) Model

The Markov chain with stationary distribution (described above) implies that the transition probabilities are independent of the time, which means that the transition probabilities of past estimation errors are theoretically the same as the transition probabilities of the present estimation errors [28]. The statistics of past estimation errors can be used as a basis for judging the errors of the new estimates [29]. In this study, the method of using RF for estimation and then using a Markov chain to estimate the error range and correct the estimation results is known as the random forest-Markov chain (RF-MC) model. The RF-MC model is applied along the following logical lines.

- It is considered that the error in RF estimation can be classified into different statuses, depending on the size.
- Assume that the estimation error of dust concentration at time *m* is related to the estimation error at time *m*−*n*, and the estimation error at time *m*−*n* is in status *a_i_*. Based on the transition probability matrix *P*(*n*), the status *a_i_* is most likely to transit to the status *a_j_* with probability *p_ij_* after time *n*. Therefore, the most probable error range at moment *m* is derived, and the estimation results are corrected.
- Since it is not certain how long the current error status has been related to the error status, consider and evaluate the current error status in relation to the status 20 s (one-step transition), 40 s (two-step transition), and 60 s (three-step transition) ago, respectively.
  Markov chain corrections also have the following characteristics.
- Markov chain corrections are based on the statistical results of a large number of known prediction errors. Moreover, the data is time-ordered, and a feature of the data can be regarded as a Markov process.
- Markov chain correction can only correct the estimations one by one in time order, unlike machine learning, where multiple sets of variables are input at once, and multiple outputs are obtained simultaneously.
  The relevant parameters are obtained, as shown in Table 1.

**Figure 4:**
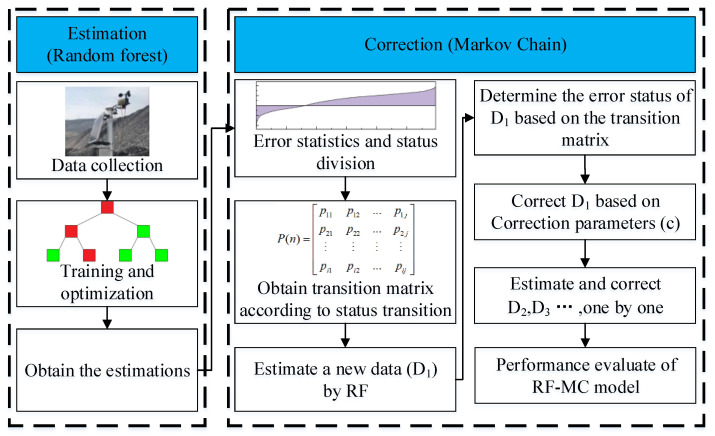
The overall flow for the RF-MC model.

The overall flow of the RF-Markov Chain Model in this study is shown in Figure 4.

## 3. Results and Discussion

### 3.1. Random Forest Estimation

From the above analysis, it is clear that the RF-MC model involves a total of three datasets: a training set for training the random forest model, a test set for testing the model, which, along with the test results, is an important dataset for establishing the transition matrix, and a correction set for testing the Markov correction performance.

Random forest model training requires a large number of datasets as a training base to ensure the applicability of the model. Moreover, data outside the model establishment process is needed as a test set for testing the performance of the model and as the base data for Markov correction. Therefore, the study must ensure that there is sufficient training data for the random forest model, as well as data for establishing the transfer matrix. As a result, 70% of the measurement data is selected as the training set and 30% as the test set. In other words, the first 7 days of the 10 days of data collected were used as the training set (29,176 data) and the last 3 days as the test set (11,905 data). The test set should normally be 12,205 data, but the study kept the last 300 data as a correction set for the Markov correction. 

Hyperparameters have an important influence on the training results of random forest models, and general machine learning research also focuses on how to determine better hyperparameters. However, the focus of this study is to analyze the ability of Markov chains to correct machine learning estimations, especially for models with poor estimation accuracy. 

Therefore, The hyperparameters in this study were chosen directly from the past training results [17]. Compared to the previous random forest model, the input for this study has no wind direction or noise and adds atmospheric pressure. As expected, a random forest model with poor estimation accuracy was obtained. The hyperparameters of RF were chosen, as shown in Table 2. 

The Pearson correlation coefficient (R), mean absolute error (MAE), and root mean squared error (RMSE) were chosen as the evaluation index for the estimation results and are calculated as follows.

(7) $$R=\frac{\sum_{i=1}^{Data}\left(x_{p}-\bar{x_{p}})(x_{r}-\bar{x_{r}}\right)}{\sqrt{\sum_{i=1}^{Data}{\left(x_{p}-\bar{x_{p}}\right)}^{2}}\sqrt{\sum_{i=1}^{Data}{\left(x_{r}-\bar{x_{r}}\right)}^{2}}}$$

(8) $$RMSE=\sqrt{\frac{1}{Data}\sum_{i=1}^{Data}{\left(x_{p}-x_{r}\right)}^{2}}$$

(9) $$MAE=\frac{1}{Data}\sum_{i=1}^{Data}\left|x_{p}-x_{r}\right|$$

*R* reflects the linear relationship between the estimated and measured values. The better the estimate, the closer the *R* is to 1. The closer it is to 0, the worse the estimate is. *RMSE* and *MAE* can directly reflect the average error between the true value *x_r_* and the estimated value *x_e_*, which is more conducive to field personnel visually judging the estimation results.

In the test set, PM2.5, PM10, and TSP were estimated, respectively, and the estimation results are shown in Table 3. Compared to the *R* values of around 0.9 obtained in a previous study, this random forest model performs poorly and has relatively large errors in the *RMSE* and *MAE*.

### 3.2. Markov Chain Correction

#### 3.2.1. Error Statistics and Transition Matrix Acquisition

Among the 12,205 data points, 11,905 estimated values were used as the training set for the Markov chain correction, and the remaining 300 retained data were used as the correction set. The error *e* is calculated according to the following formula.

(10) $$e=\frac{x_{e}-x_{r}}{x_{e}}$$

**Figure 5:**
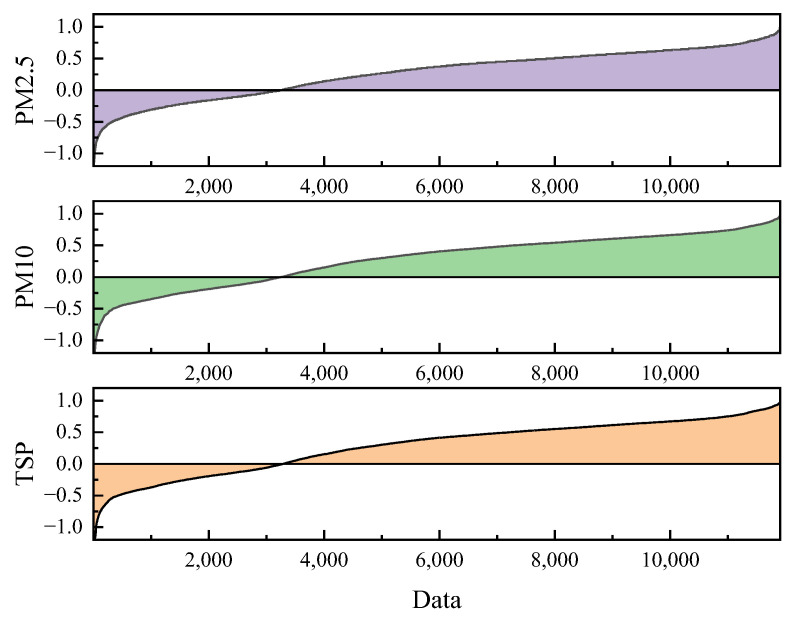
Estimation error distribution.

Figure 5 shows the estimation error distribution for 11,905 estimated values.

The PM2.5, PM10, and TSP errors are mainly distributed between −1 and 1. The middle of the range of error status is used as the correction parameter *c*, so the greater the number of statuses, the smaller the maximum possible correction error when the status estimate is accurate. In this study, the error *e* was divided into 16 levels, as shown in Table 4.

The following equation is the formula for the Markov chain correction for the corrected data *x_mc_*.

(11) $$x_{mc}=x_{e}(1-c)$$

The error status was divided for all data in the training and correction sets. The transition status of the training set data was determined according to the transition step *n* = 1, 2, 3, respectively. Table 5 shows the status transition of some of the data for PM2.5. The transition statuses derived from the different transition steps for PM2.5, PM10, and TSP were then counted separately. Table 6 shows the one-step transition matrix for PM2.5. After obtaining the transition matrix for the different transition steps, the RF was applied to the 300 corrections set data. Afterwards, each estimated data was corrected in time order.

#### 3.2.2. Result Correction of RF Estimation

The *RMSE* comparison of the estimated values, before and after the Markov chain correction for the 300 data, is shown in Table 7. The estimated values after the Markov correction are significantly better than those before the correction, with the one-step transition correction being the best, indicating that the estimated data status is the most relevant to the previously estimated data error.

**Figure 6:**
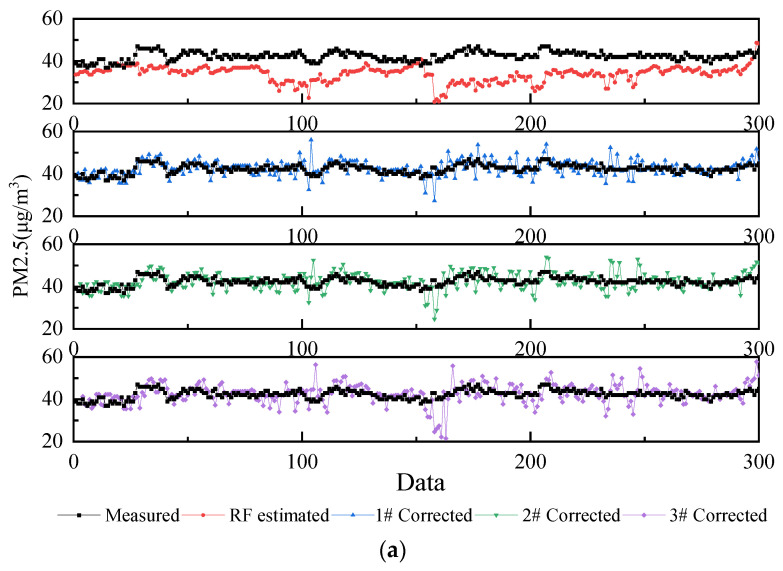

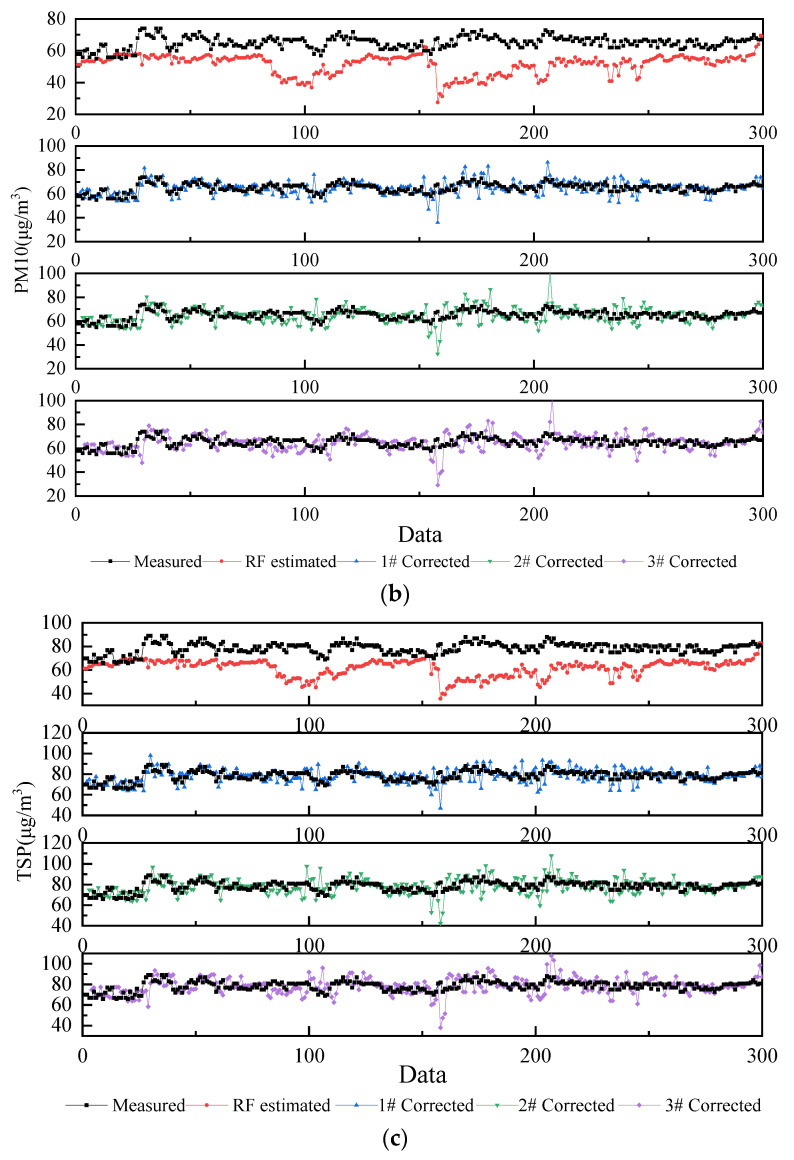
Comparison of curves before and after correction: (**a**) comparison curve of PM2.5; (**b**) comparison curve of PM10; (**c**) comparison curve of TSP.

The measured values from the field, the estimated values by RF, and the corrected values by Markov chain are shown in Figure 6, where 1#, 2#, and 3# represent a one-step transition, two-step transition, and three-step transition, respectively. The fold line shows some error between the measured data and the estimated data, with the estimated data being small overall. The *RMSE* and *MAE* of the corrected data are significantly smaller. The corrected curve is overall near the measured data curve. However, there are fluctuations caused by over-correction or under-correction, and this fluctuation is also reflected in the R-value. The possible reasons for this may be that the noise of the measured data affects the transition probability, that the number of status divisions is insufficient, or both.

**Figure 7:**
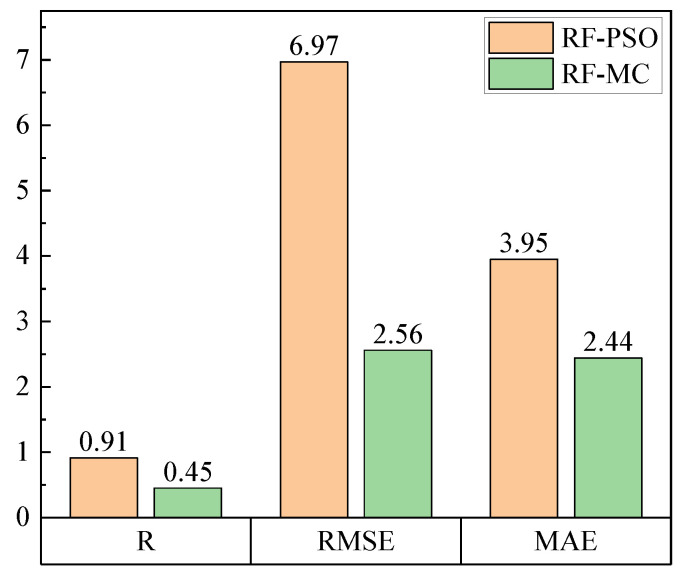
Comparison of the two performance evaluations.

As seen in Figure 7, in the previous study, the R in the PM2.5 performance evaluation reached 0.91, far better than the 0.45 performance in this study. Although the RMSE was reduced from 6.97 to 2.56 and the *MAE* from 3.95 to 2.44, the inconsistency in the range of dust concentrations between the two studies makes it difficult to compare the *RMSE* and *MAE*.

Although the corrected *R* is still not good enough, the performance of *RMSE* and *MAE* is more important for field applications. The 1st level limits of the daily average for PM2.5, PM10, and TSP required in China are 0–35, 0–50, and 0–120, respectively, while the *RMSE* and *MAE* for TSP are only 6.27 and 4.28. The accuracy of the estimates in this study will not cause a large bias in determining dust concentrations in the field. Similar conclusions were drawn for PM2.5 and PM10. In addition, it can also be found that the larger the dust particle size, the worse the estimation. Future research on PM10 and TSP may need to consider more features or different approaches in order to further improve the estimation effect. Overall, Markov chains can simply and significantly improve the estimation accuracy of random forest models with poor accuracy to a level that can be applied in the field.

### 3.3. Discussion of the Feasibility of Application in Chinese Open-Pit Coal Mines

**Figure 8:**
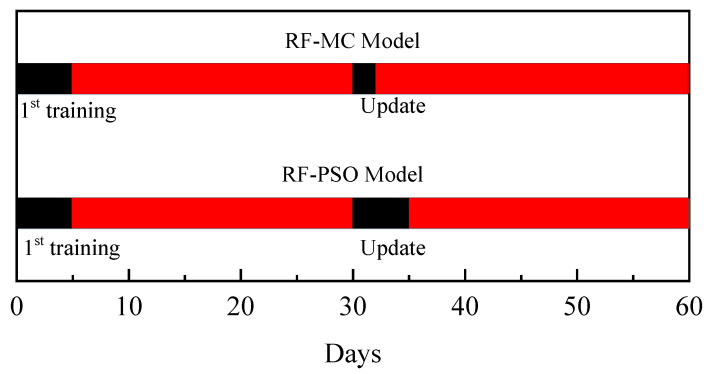
Application comparison of RF-PSO model and RF-MC model.

The essence of the RF-PSO model is that RF updates the model, while PSO improves the speed with which the RF model is updated. Due to the excellent correction effect of the MC model, the RF-MC model update is mainly an update of the MC model. The RF model update frequency can be very low as long as the estimation accuracy after MC correction can meet the field requirements. At the same time, MC model updates are statistically more easily and quickly performed than those for the RF-PSO model, and they also have the potential for automation. This makes learning and maintaining estimation models easier for open-pit mine managers. A schematic comparison of the two models is shown in Figure 8, where a specific update interval is assumed for the model.

For input parameter acquisition, estimation models require parameters such as temperature and humidity as inputs. Although these inputs need to be estimated, new energy policies in China in recent years have opened the possibility of obtaining these inputs for open-cast coal mines [30]. To ensure a stable supply of electricity and a higher proportion of new energy, the Chinese government has in recent years been promoting the construction of energy bases, i.e., thermal power plants near coal mines, to ensure the supply of coal. At the same time, photovoltaic power stations are being built in the dumps of open-pit coal mines, and wind power plants are being built nearby, where meteorological conditions permit. In order to ensure that the proportion of electricity generated by different energy sources is regulated adequately at different times, the local meteorological authorities are required to provide detailed weather forecasts to the energy bases or to set up small weather forecasting stations at the energy bases, which provide reliable input data—such as temperature and humidity—needed to estimate dust concentrations.

Regarding sprinkler dispatch, there are two main levels. The first is to estimate the overall daily dust concentration and prepare the number of sprinklers needed to be deployed in advance. Second, it takes approximately one hour for the mine’s sprinklers to prepare to start sprinkling at the designated location, so estimating every hour of the day means that the sprinklers can be scheduled consistently, while parameters such as temperature and humidity are announced by the meteorological office at one-hour intervals.

Concerning the overall open-pit coal mine application, there are inconsistencies in dust pollution levels in different production areas due to different production intensities, meaning that the model built based on data from one monitoring point may not be suitable for estimating other monitoring point areas. Therefore, the adaptability of different monitoring areas to a single model should also be tested in future studies or field applications. Potentially, multiple estimation models may be needed to satisfy the demands different areas. Moreover, model reliability during seasonal transitions is being tested to determine whether there is an impact on model accuracy when large changes in climate occur.

In addition, this study is also an essential contribution to green and climate-smart mining. In China, the concept of green mining started in 2003 [31] and has been strongly endorsed by the government and society [32]. It remains the development objective of the Chinese coal industry [33]. Green mining utilizes green technologies to improve environmental efficiency and maintain the mining industry’s competitiveness over a mine’s entire life cycle. It is particularly crucial for supplying minerals in a way that is economically feasible, socially advantageous, and environmentally responsible [34]. As an integral component of green open-pit mining, dust management is critical for reducing the environmental pollution in and around mines to protect human health and operation safety [35]. For example, Table 8 showing China’s National Ambient Air Quality Standards (GB3095-2012), in conjunction with Figure 2a, indicates that the PM2.5 and PM10 concentrations are at the 2nd level for several time intervals, causing health and safety problems, environmental penalties, and loss of production. The accurate estimation of dust concentration not only provides technical support for road dust management, but also offers directions for managing dust in other operations, such as drilling, blasting, and crushing station unloading. By accurately estimating dust concentrations, our proposed approach assists inherently dust-producing open-pit mining operations in ensuring the sustainable development of mine operations and realizing green and climate-smart mining.

## 4. Conclusions

Dust management in open-pit mines is critical to ensuring miners’ health, the safety of production operations, and the reduction of environmental pollution, which are key components of China’s green mining development. This study proposes an RF-MC model to estimate dust concentrations in open-pit coal mines with an *RMSE* of only 2.56 (PM2.5), 5.28 (PM10), and 6.27 (TSP), which meets the requirements of estimation accuracy and field application, while also providing technical support for the rational utilization of water resources and dust management in mines. The findings showed that the correction process of the Markov chain is relatively simple. It mainly involves error statistics, status classification, and status transfer statistics. It is faster and simpler than training random forests, making it more suitable for application in open-pit coal mines. Meanwhile, relying on Markov chains can improve the accuracy of random forest models with poor accuracy. After the one-step transition matrix correction, the *RMSE* of PM2.5 reduced from 7.40 to 2.56, of PM10 from 15.73 to 5.28, and of TSP from 18.99 to 6.27. In comparison, the two and three-step transition matrix corrections are slightly less effective, but still noticeable. The improved machine learning method proposed in the study uses Markov chain correction as its core. In the first RF-MC model application, the training is mainly for RF. Each time the model accuracy decreases afterwards, a rapid error correction is performed, using only MC, to update the model accuracy and bring it back into use. 

In future research, the influence of time and space on the accuracy of the dust estimation model will be one of the study directions in order to ensure the performance of the estimation model for its application over the whole open-pit coal mine.

## Figures and Tables

**Table 1:** Relevant parameters of the Markov chain.

Parameters	Symbols	Methods of Access
Status	*a*_1_, *a*_2_, *a*_3_, ...	Determined by the error distribution of the previous estimation results
Transition probability	*p*_11_, *p*_12_, *p*_22_...	Statistics on the error status transition of the estimation results

**Table 2:** Hyperparameters of the random forest.

Hyperparameter	PM2.5	PM10	TSP
Max depth	15	14	13
Number DT	186	114	913
Min samples split	14	2	2
Min samples leaf	7	4	2
Max features	0.4027	0.4176	0.4007

**Table 3:** Random forest estimation results.

Index	PM2.5	PM10	TSP
*R*	0.37	−0.36	−0.37
*RMSE*	15.62	23.85	29.34
*MAE*	13.32	20.69	25.44

**Table 4:** Status classification of estimation error.

Status	Range	Correction Parameters (*c*)
*a*_1_	*e* < −0.875	−0.9375
*a*_2_	−0.875 ≤ *e* < −0.75	−0.8125
*a*_3_	−0.75 ≤ *e* < −0.625	−0.6875
*a*_4_	−0.625 ≤ *e* < −0.5	−0.5625
*a*_5_	−0.5 ≤ *e* < −0.375	−0.4375
*a*_6_	−0.375 ≤ *e* < −0.25	−0.3125
*a*_7_	−0.25 ≤ *e* < −0.125	−0.1875
*a*_8_	−0.125 ≤ *e* < 0	−0.0625
*a*_9_	0 ≤ *e* < 0.125	0.0625
*a*_10_	0.125 ≤ *e* < 0.25	0.1875
*a*_11_	0.25 ≤ *e* < 0.375	0.3125
*a*_12_	0.375 ≤ *e* < 0.5	0.4375
*a*_13_	0.5 ≤ *e* < 0.625	0.5625
*a*_14_	0.625 ≤ *e* < 0.75	0.6875
*a*_15_	0.75 ≤ *e* < 0.875	0.8125
*a*_16_	*e* ≥ 0.875	0.9375

**Table 5:** The status transition of some of the data for PM2.5.

Estimated	Measured	Error	Status	1-Step Transition	2-Step Transition	3-Step Transition
27.65475	38	−0.3741	6			
23.22106	38	−0.6364	3	6, 3		
24.99318	37	−0.4804	5	3, 5	6, 5	
27.40549	37	−0.3501	6	5, 6	3, 6	6, 6
25.67323	38	−0.4801	5	6, 5	5, 5	3, 5
23.71703	38	−0.6022	4	5, 4	6, 4	5, 4
23.53559	38	−0.6146	4	4, 4	5, 4	6, 4
18.94361	39	−1.0587	1	4, 1	4, 1	5, 1
20.73295	39	−0.8811	1	1, 1	4, 1	4, 1
20.22708	38	−0.8787	1	1, 1	1, 1	4, 1
21.25913	38	−0.7875	2	1, 2	1, 2	1, 2
19.6952	39	−0.9802	1	2, 1	1, 1	1, 1
19.6712	38	−0.9318	1	1, 1	2, 1	1, 1
21.01195	38	−0.8085	2	1, 2	1, 2	2, 2
19.88806	40	−1.0113	1	2, 1	1, 1	1, 1

**Table 6:** One-step transition matrix for PM2.5. (The % is omitted after the number).

Status	*a*_1_	*a*_2_	*a*_3_	*a*_4_	*a*_5_	*a*_6_	*a*_7_	*a*_8_	*a*_9_	*a*_10_	*a*_11_	*a*_12_	*a*_13_	*a*_14_	*a*_15_	*a*_16_
*a*_1_	0.09	0.07	0.04	0.04	0.02	0.02	0.00	0.02	0.00	0.02	0.01	0.00	0.01	0.00	0.00	0.00
*a*_2_	0.05	0.08	0.04	0.05	0.03	0.03	0.02	0.00	0.00	0.00	0.00	0.00	0.00	0.00	0.00	0.00
*a*_3_	0.05	0.06	0.15	0.13	0.07	0.11	0.05	0.01	0.01	0.00	0.00	0.00	0.00	0.00	0.00	0.00
*a*_4_	0.03	0.03	0.13	0.48	0.47	0.17	0.08	0.05	0.03	0.01	0.01	0.00	0.00	0.00	0.00	0.00
*a*_5_	0.02	0.02	0.10	0.39	1.31	0.85	0.27	0.15	0.03	0.01	0.01	0.02	0.00	0.00	0.00	0.00
*a*_6_	0.03	0.02	0.08	0.17	0.82	2.34	1.24	0.37	0.13	0.07	0.01	0.00	0.00	0.00	0.00	0.00
*a*_7_	0.02	0.01	0.03	0.14	0.25	1.26	4.64	1.54	0.32	0.05	0.03	0.01	0.00	0.00	0.00	0.00
*a*_8_	0.02	0.01	0.03	0.06	0.09	0.34	1.61	4.28	1.04	0.32	0.06	0.02	0.03	0.00	0.00	0.00
*a*_9_	0.01	0.01	0.01	0.01	0.08	0.09	0.17	1.13	2.24	1.33	0.28	0.07	0.02	0.01	0.00	0.00
*a*_10_	0.01	0.01	0.01	0.03	0.05	0.13	0.22	1.31	4.14	1.60	0.40	0.07	0.00	0.00	0.00	0.00
*a*_11_	0.01	0.00	0.00	0.00	0.00	0.01	0.07	0.10	0.23	1.61	4.75	2.55	0.45	0.03	0.00	0.00
*a*_12_	0.00	0.00	0.01	0.00	0.01	0.00	0.00	0.02	0.05	0.34	2.49	9.63	3.01	0.34	0.03	0.00
*a*_13_	0.00	0.00	0.00	0.00	0.00	0.00	0.01	0.03	0.03	0.06	0.48	2.97	9.48	3.28	0.14	0.00
*a*_14_	0.00	0.01	0.00	0.01	0.00	0.00	0.01	0.00	0.02	0.01	0.08	0.24	3.27	7.21	0.97	0.08
*a*_15_	0.00	0.00	0.00	0.00	0.00	0.00	0.00	0.00	0.00	0.01	0.02	0.02	0.15	0.92	2.77	0.33
*a*_16_	0.00	0.00	0.00	0.00	0.00	0.00	0.00	0.00	0.00	0.00	0.00	0.00	0.00	0.10	0.31	0.49

**Table 7:** Comparison before and after Markov correction.

	Index	RF Estimated	1# Corrected	2# Corrected	3# Corrected
PM2.5	*R*	−0.13	0.45	0.36	0.33
	*RMSE*	7.40	2.56	2.99	3.51
	*MAE*	8.51	2.44	2.97	3.27
PM10	*R*	−0.17	0.47	0.31	0.26
	*RMSE*	15.73	5.28	6.62	7.17
	*MAE*	13.73	3.98	4.98	5.43
TSP	*R*	−0.16	0.45	0.32	0.29
	*RMSE*	18.99	6.27	7.62	8.15
	*MAE*	16.75	4.78	5.83	6.23

**Table 8:** Limits of the daily average of particulate matter concentrations.

Particulate Matter	Average Time	Concentration Limit (μg/m^3^)	
		1st Level (Good Air Quality)	2nd Level (Impact on Health)
PM2.5	Daily average concentration	0~35	36~75
PM10		0~50	51~150
TSP		0~120	121~300

## Data Availability

Not applicable.
